# Genetic Counselors’ Perspectives and Practices Regarding Expanded Carrier Screening after Initial Clinical Availability

**DOI:** 10.1007/s10897-015-9881-1

**Published:** 2016-04-01

**Authors:** Gabriel A. Lazarin, Stacey Detweiler, Shivani B. Nazareth, Elena Ashkinadze

**Affiliations:** 1 Counsyl, 180 Kimball Way, South San Francisco, CA 94080 USA; 2 Department of Maternal-Fetal Medicine, Rutgers-Robert Wood Johnson Medical School, New Brunswick, NJ USA

**Keywords:** Expanded carrier screening, Recessive disease, Survey, Genetic counselor, Reproductive genetics, Beliefs, Attitudes, Clinical practice

## Abstract

Expanded carrier screening (ECS), introduced in 2009, identifies carriers for dozens or hundreds of recessive diseases. At the time of its introduction into clinical use, perspectives of the genetic counseling community regarding ECS were unknown. We conducted a survey in early 2012 of GCs and report the results here. They represent a snapshot of opinions and usage at that time, providing a baseline for comparison as the technology continues to evolve and as usage increases. The survey assessed personal perspectives, opinions on clinical implementation and clinical utilization of ECS. The sample included 337 GCs of varying clinical fields, of whom 150 reported practicing in reproductive settings. Our findings demonstrate that, at the time, GCs indicated general agreement with ECS as a concept – for example, most GCs agreed that carrier screening should address diseases outside of current guidelines and also indicated personal interest in electing ECS. There were also disagreements or concerns expressed regarding appropriate pre- and post-test counseling (e.g., the content and delivery mode of adequate informed consent) and practical implementation (e.g., the amount of time available for follow-up care). This was the first quantitative study of a large number of GCs and it revealed initial overall support for ECS among the GC profession. The authors plan to re-administer a similar survey, which may reveal changes in opinions and/or utilization over time. A follow up survey would also allow further exploration of questions uncovered by these data.

## Introduction

Expanded carrier screening (ECS) uses next-generation sequencing or microarray hybridization analysis to detect mutations in many genes associated with recessive genetic diseases. The same objective remains as that of standard single-gene analysis protocols: identification of couples at risk for transmitting genetic conditions in order to guide reproductive decision-making in the prenatal or preconception period (Edwards et al. 2015; Grody et al. 2013). Clinically introduced in 2009, multiple laboratories now offer panels that vary in diseases and mutations tested and other characteristics. The general focus, however, tends toward diseases with pediatric implications. By comparison, carrier screening has traditionally focused on limited diseases of high prevalence, either in certain ethnicities or universally, based on guidelines issued by the American Congress of Obstetricians and Gynecologists (ACOG) and American College of Medical Genetics and Genomics (ACMG) (ACOG 2007, 2009, 2011; Gross et al. 2008; Prior 2008).

Carrier screening is voluntary in nature. Though individuals may decide whether to undergo screening, the specific testing options available to them are often dependent on the provider’s practices, since a physician’s offering and prescription are generally required. Given the lack of guidance on whether and how to offer ECS, varying clinical practices can be anticipated.

Genetic counselors (GC) are exposed to and may utilize emerging genetic testing technologies more rapidly. Therefore, their insights and preferences regarding expanded screening often influence other providers who offer carrier screening. Limited data are available regarding GC attitudes, beliefs and preferences regarding ECS. Cho et al. (2013) conducted focus group interviews and identified themes of perceived benefits and disadvantages of ECS. For example, their participants expected that patient interest in ECS would be high and acknowledged its financial value, but they also expressed concern about the possibility of false reassurance and specific construction of the disease panels. Forty genetics professionals participated in their study, a sample which may have included non-GCs. Therefore, the Cho et al. study represents a small subset of the total GC population (National Society of Genetic Counselors 2012).

This study’s purpose was to conduct an extensive survey in a large GC population on personal and professional attitudes regarding ECS. To our knowledge, this was the first study of its kind.

## Methods

### Sample and Procedures

An anonymous, online survey assessing knowledge and attitudes of GCs toward ECS was distributed to all 3,039 participating members of the National Society of Genetic Counselors (NSGC) via email from 2/2012 to 4/2012 (NSGC 2012). By consenting to participate, responders verified that they held a master’s degree in Genetic Counseling or Human Genetics, or equivalent. The survey was anonymous but participants could voluntarily enter a raffle for one of three iPads (Apple Corporation, Cupertino, CA). We utilized an online survey service, Wufoo (SurveyMonkey, Inc., Palo Alto, CA). This study was approved by the Institutional Review Board of the University of Medicine and Dentistry of New Jersey, now Rutgers-Robert Wood Johnson Medical School (New Brunswick, NJ).

### Instrumentation

The investigator-created survey consisted of 65 questions divided into 7 sections. Sections included were: criteria for inclusion in an ECS panel (9 questions), GCs’ personal feelings about ECS (9 questions), experience with ECS tests (6 questions), role of genetic counseling for ECS (8 questions), criteria for offering ECS (19 questions), knowledge assessment (7 questions) and demographics (7 questions). The majority of questions evaluated level of agreement using a 5-point Likert scale. Since all survey questions were voluntary, a fluctuation in question-specific response rate was possible.

### Data Analysis

Frequencies were calculated for responses to each of the survey items. On some items, we also compared responses between participants working in reproductive genetics settings and those working in others. We assessed statistical significance by utilizing the pooled two sample z-test.

## Results

### Response Rate and Demographics

In total, 337 GCs completed the survey, resulting in an 11.1 % response rate based on the 2012 NSGC membership of 3,039. Forty-four percent (*n* = 150) of the respondents indicated reproductive genetics as their primary field of practice, meaning either prenatal, infertility or ART/IVF, or PGD/preconception settings were their primary practice types. In 2012, NSGC reported approximately 900 GCs working in reproductive fields (prenatal, infertility, ART/IVF, and PGD/preconception) (NSGC 2012). Therefore, we estimated that we surveyed approximately 17 % of reproductive GCs. Our study sample was 94 % female and 91 % Caucasian, consistent with the NSGC membership demographics at the time (NSGC 2012). The majority (61 %) were 25–34 years old. Geographically, the highest representations came from Regions II (34 %), consisting of states from New York to the Mid-Atlantic, and IV (25 %), which encompasses the Midwest. Almost half of respondents (48 %) had 1–4 years of genetic counseling experience, and 25 % had 5–9 years of experience. Various work settings were reported, including university medical center (37 %), private hospital/medical facility (20 %), public hospital/medical facility (17 %), commercial laboratory (8 %), and physician private practice (7 %). Table 1 reports complete demographic information.

**Table 1:** Responder demographics^a^

	Reproductive GC n (%)	Non-reproductive GC n (%)
Gender		
male	6 (4.3 %)	14 (7.5 %)
female	133 (95.7 %)	174 (92.6 %)
Age		
20–24	5 (3.7 %)	8 (4.4 %)
25–29	45 (33.1 %)	70 (38.0 %)
30–34	30 (22.1 %)	51 (27.7 %)
35–39	30 (22.1 %)	30 (16.3 %)
40–44	9 (6.6 %)	12 (6.5 %)
45–49	10 (7.3 %)	5 (2.7 %)
50–54	7 (5.2 %)	8 (4.4 %)
Ethnicity		
American Indian or Alaskan Native	0	1 (0.5 %)
Asian	5 (3.6 %)	13 (6.6 %)
Black or African-American	2 (1.4 %)	1 (0.5 %)
Caucasian or white	133 (94.3 %)	184 (88.8 %)
Hispanic/Chicano/Latino	0	4 (2.0 %)
Native Hawaiian or Pacific Islander	0	2 (1.0 %)
other	1 (0.7 %)	1 (0.5 %)
NSGC region		
Region 1 (CT, MA, ME, NH, RI, VT, CN Maritime Provinces)	12 (8.5 %)	15 (8.0 %)
Region 2 (DC, DE, MD, NJ, NY, PA, VA, WV, PR, VI, Quebec)	45 (31.7 %)	66 (35.0 %)
Region 3 (AL, FL, GA, KY, LA, MS, NC, SC, TN)	10 (7.0 %)	14 (7.4 %)
Region 4 (AR, IA, IL, IN, KS, MI, MN, MO, ND, NE, OH, OK, SD, WI, Ontario)	32 (22.5 %)	51 (27.0 %)
Region 5 (AZ, CO, MT, NM, TX, UT, WY, Alberta, Manitoba, Sask.)	21 (15.0 %)	18 (9.5 %)
Region 6 (AK, CA, HI, ID, NV, OR, WA, British Columbia)	22 (15.5 %)	25 (13.2 %)
Years in practice		
1–4	64 (51.2 %)	88 (49.7 %)
5–9	25 (20.0 %)	54 (30.5 %)
10–14	26 (20.8 %)	31 (17.5 %)
15–19	10 (8.0 %)	4 (2.3 %)
20–25	0	0
> 25	5 (4.0 %)	9 (5.1 %)

^a^: *n* = 337 total GCs completed the survey by reaching its end. Individuals were not required to answer every question. Throughout the survey, and in this table, responses may not sum to 337

Although we surveyed GCs from all fields in order to gain broad perspectives and potentially observe differences, some results below pertain only to those who indicated current practice in reproductive genetics (*n* = 150). In general, where questions pertained to actual clinical practice, we report data only from GCs that reported working in reproductive genetics (RGCs). Where questions assess opinions or beliefs, we report all pooled data. Though in general there were no differences between GCs and RGCs, key areas of statistically significant differences are described at the end of the Discussion.

### Genetic Counselor Personal Perspectives

**Fig. 1:**
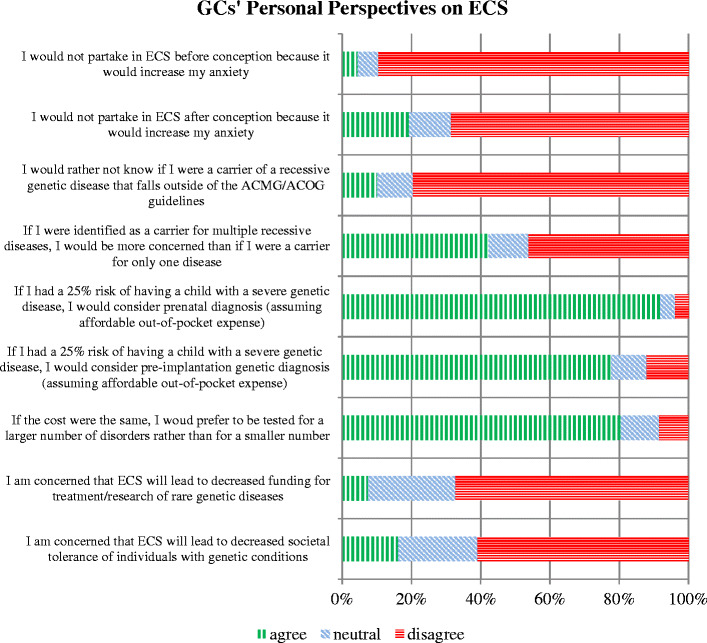
GCs’ personal perspectives on ECS

Personal perspectives were ascertained with the recognition that what a GC may choose for him/herself may differ from what is offered to patients in a clinical setting. Among all GCs surveyed, most reported that they would personally pursue ECS and would rather be screened for a larger number of conditions. A minority, 19 %, would opt out of ECS during pregnancy and fewer (5 %) would opt out prior to conception due to anxiety. The majority (80 %) agreed that if cost were the same, they would prefer to be tested for a large number of conditions – and 90 % would want to know if they were carriers for conditions beyond the ACOG/ACMG guidelines. When considering reproductive options for carrier couples, 92 % would personally opt for prenatal diagnosis (CVS or amniocentesis) if the fetus were at risk for a recessive condition. Interestingly, fewer (78 %) would consider pre-implantation genetic diagnosis to reduce their reproductive risks. The majority disagreed with the statements that ECS would lead to decreased funding for genetic disorders (67 %) or decreased societal tolerance of disabilities (61 %). Refer to Fig. 1 for comprehensive response data for this section.

### Clinical Availability and Implementation

**Fig. 2:**
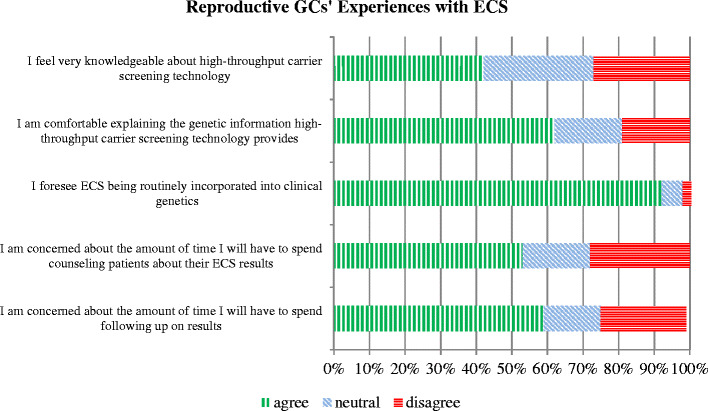
Reproductive GCs’ experiences with ECS

We report reproductive GCs (RGCs) views on early clinical availability of ECS and its implementation into clinical practice. Less than half (42 %) of RGCs responded that they were very knowledgeable about ECS technology. However, 62 % were comfortable explaining the genetic information ECS would provide (Fig. 2). The majority did not offer ECS at all (52 %) and (40 %) offered it to less than one-third of their patients. Six RGCs were offering ECS to all of their patients, but 92 % stated ECS would be routinely incorporated into clinical genetics in the future. Some of the barriers to routine implementation included concerns such as amount of time spent counseling patients regarding ECS results (53 %) and time needed to coordinate follow-up testing (59 %).

Two questions assessed the potential impact of perceived socioeconomic status (SES) on the implementation of ECS in clinical practice: *I use my perception of the patient’s SES in choosing which test options I offer*; and *I offer all of my patients identical carrier screening options, regardless of their SES.* We expected responses to these two questions to be consistent. Consistency would be observed if responders disagreed with the first statement and agreed with the second – effectively, saying, “I *do not* use perceived SES in choosing which tests to offer, therefore I *do* offer all of my patients identical carrier screening options regardless of SES.” RGCs generally disagreed (85 %) with the first statement, but less agreed with the second (74 %). While the discrepancy only applies to a minority of respondents, it was statistically significant (*p* = 0.03).

### Perspectives on Panel Inclusion

**Fig. 3:**
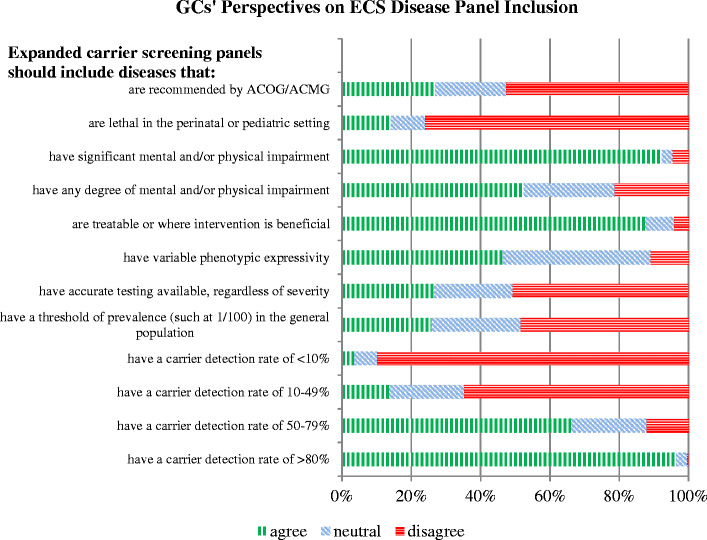
GCs’ perspectives on ECS disease panel inclusion

Another survey goal was to assess views on diseases to be included on an ECS panel. These data regard opinions on all GCs surveyed. Of all respondents, 27 % agreed that ECS should be limited to conditions recommended by ACMG/ACOG for general population screening. When assessing clinical severity as a criterion for disease inclusion, 14 % felt that conditions should be limited to those that are lethal in the neonatal period. The majority (92 %) agreed that conditions with significant physical and/or mental impairment should be included in ECS, while 52 % agreed that a condition with any degree of impairment should be included. Eighty-eight percent stated ECS should include conditions which are treatable or in which intervention is beneficial. Approximately 11 % of respondents disagreed with including conditions that have variable phenotypic expressivity. Regarding the statement, “Expanded panels should include any autosomal recessive condition for which accurate testing is available, regardless of severity,” 51 % disagreed and 27 % agreed, with the remainder being neutral. Forty-nine percent disagreed that assessing disease prevalence should be used as a criterion for disease inclusion. The majority (90 %) agreed that conditions assessed with a detection rate of less than 10 % should not be included in the panel. However, 66 % agreed that a detection rate of above 50 % is acceptable and almost all respondents (96 %) stated that a condition with a >80 % detection rate may be included. Refer to Fig. 3 for comprehensive response data.

This survey section contained the only two questions that revealed statistically different responses between RGCs and non-RGCs. If not working in the reproductive field, a GC was more likely to respond that diseases of any severity should be included in an ECS panel (32 % versus 21 %, *p* = 0.02). Also, if not working in a reproductive field, a GC was less likely to agree that there should be a minimum prevalence threshold in order to include a disease on an ECS panel (22 % versus 31 %, *p* = 0.04).

### The Role of the Genetic Counselor in the Service Delivery Model of ECS

**Figure 4:**
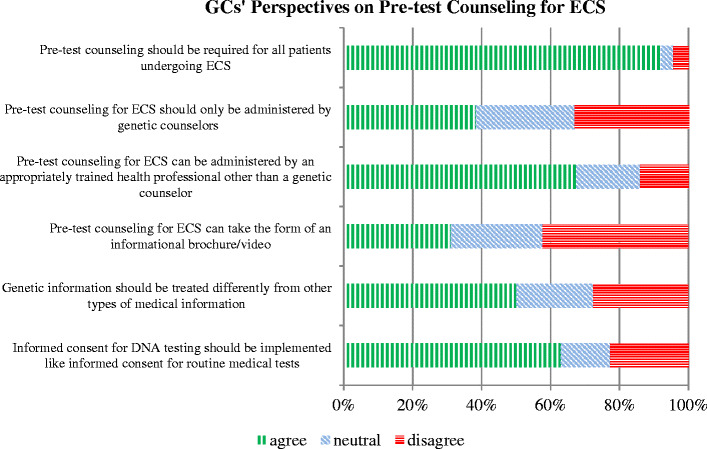
GCs’ perspectives on pre-test counseling for ECS

Perspectives on the GCs role in criteria for offering ECS, and the role of GCs in its delivery, were evaluated. Among all respondents, 92 % stated that pre-test counseling should be required for all patients prior to having ECS. However, 67 % agreed that properly trained health professionals other than GCs could administer pretest counseling, and 31 % stated that pre-test counseling could be in the form of an information brochure or video (refer to Fig. 4).

We assessed opinions on the necessary components of informed consent for ECS. Options were to group diseases by severity, type (e.g., neurological), prevalence, ethnic predilection, all diseases should be described individually or that generic consent model was appropriate. Multiple responses could be chosen. The two most common responses were: a generic consent model (*n* = 80), or diseases should only be presented in groups by severity (*n* = 69). This accounts for all possible responses, including multiple selections (Table 2). Note that statements on ECS from professional societies, which assert sufficiency of a generic consent model, were not yet published at the time of this survey (Edwards et al. 2015; Grody et al. 2013).

**Table 2:** Pre-test ECS presentation, most common responses

Presentation element(s)	Agreements, n
ECS is sufficiently presented by saying that it identifies diseases that can cause a wide range of complications (generic model).	80
Categorized by severity (e.g., lethal, treatable)	69
Categorized by severity, and Type (e.g., neurological), and Prevalence (e.g., most common, very rare), and Ethnic predilection	43
Categorized by severity, and Prevalence, and Ethnicity	36

We also surveyed participants regarding results scenarios that merit post-test counseling (Table 3): all results, one partner positive, both partners positive, only by request, or none. Multiple options could be chosen. The most common response was that three scenarios indicate post-test counseling: when one partner is positive, when both are positive or upon request (*n* = 148). The second most common response was that all results should be counseled (*n* = 63).

**Table 3:** Post-test ECS counseling, most common responses

Counseling indication(s)	Agreements, n
One partner positive carrier status, or Both partners positive carrier status, or On request	217
Always, regardless of results	84
One partner positive carrier status	55
Both partners positive carrier status, or On request	36

**Figure 5:**
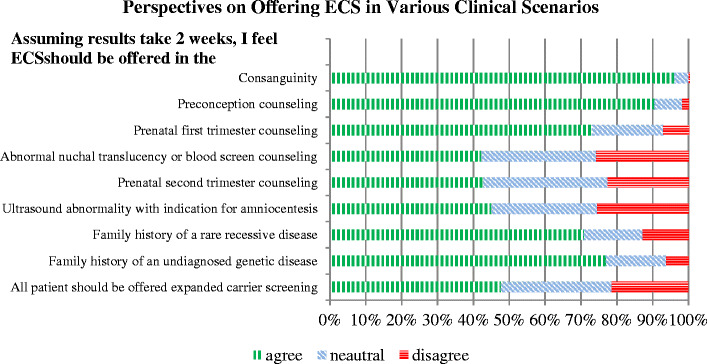
Perspectives on offering ECS in various clinical scenarios

The last set of questions surveyed clinical and non-clinical factors that influence offering ECS. Nearly half of all GCs (48 %) agreed that all patients should be offered ECS. We asked, assuming a 2 week results turnaround time, in which clinical circumstances should ECS be offered: 96 % agreed in the context of consanguinity, 90 % agreed offering pre-conceptionally, 75 % agreed offering ECS prenatally, 71 % would offer if there were a family history of a rare recessive disorder and 77 % would offer if there were a family history of an undiagnosed genetic disease. Refer to Fig. 5 for comprehensive response data.

**Figure 6:**
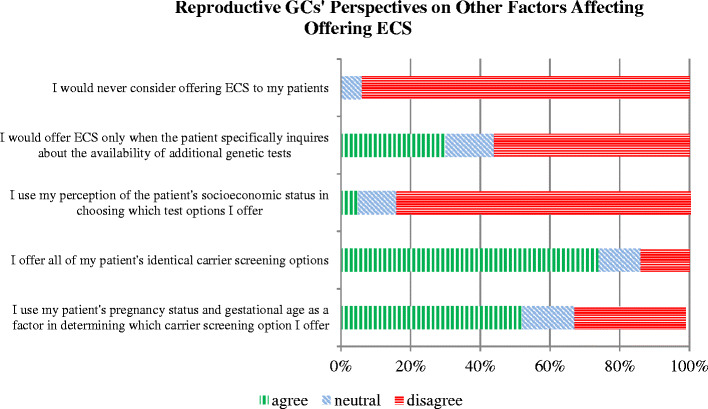
Reproductive GCs’ perspectives on other factors affecting offering ECS

Regarding non-clinical factors that affect likelihood of offering ECS, 30 % of RGCs would *only* offer ECS if a patient inquired about the availability of additional genetic testing and 90 % would offer ECS if any patient requests as much information as possible. Although ECS panels incur costs relatively similar to those of single-gene carrier screens, we attempted to assess whether perceived financial status may affect whether a patient is offered this new technology. When asked whether they offer all patients identical carrier screening, regardless of socioeconomic status, 74 % agreed and 14 % did not. A minority (5 %) stated that they use perceived socioeconomic status in choosing which testing options to offer. Refer to Fig. 6 for comprehensive response data.

## Discussion

The objective of this study was to assess GCs’ personal and clinical perspectives on ECS. The survey was administered in April 2012, approximately 2 years after initial clinical availability. There has since been substantial growth in utilization and in recognition: Counsyl’s ECS testing volume has grown nearly 400 % from April 2012 to March 2015 (unpublished data) and a comprehensive statement was issued in February 2015 by ACOG, ACMG, NSGC, the Society for Maternal-Fetal Medicine and the Perinatal Quality Foundation. Therefore, these data offer a representation of early perspectives and establish a basis for comparison for later surveys. We plan to re-administer a similar survey, with potential for elaboration on some of the questions raised in this study.

### Support for Expanded Carrier Screening; Concerns for Implementation

We found support for expanded carrier screening in this GC population, including those practicing in reproductive settings. Several questions assessed its desirability or appropriateness and three data points most simply illustrate this support: 1) only 27 % of all GCs responded that screening panels should be limited to those diseases currently addressed by ACMG/ACOG guidelines; 2) given equal cost, 80 % would personally elect expanded screening; and 3) 92 % expect ECS to become routine practice. In summary, the group largely accepted the ECS premise, despite lack of professional society endorsement, for personal utilization or for current or future practice.

Cho, et al. (2013) summarized their focus group findings and found both support and caution regarding ECS. Since those responses were not quantified, our results are not directly comparable. In comparison to other health care professionals, though, our findings are consistent with ECS acceptance described in surveys of ACOG and American Society of Reproductive Medicine audiences (Benn et al. 2014; Ready et al. 2012).

However, respondents identified questions or concerns with regard to practical implementation, also consistent with the same previous studies (Benn et al. 2014; Ready et al. 2012). Half of RGCs stated concern regarding time spent counseling ECS results or related follow-up testing. Regarding pre-test informational conveyance, the most common responses were a generic consent model or to present diseases according to levels of severity. However, as yet, there is no consensus on ascribing severity levels to the broad swath of diseases that can be included on an ECS panel, though a potential solution has been recently proposed (Lazarin et al. 2014a). A conclusion, though, is that given the many details that are possible to discuss before testing, the most common responses focused on simplicity rather than inclusion of all information. A statement by the ACMG and separate joint statement by multiple organizations, released after this survey, propose a generic pre-test approach via informational brochure or video, with genetic counseling available upon request (Edwards et al. 2015; Grody et al. 2013).

Insufficient numbers of GCs in the US and elsewhere is a known limitation – there were approximately four million pregnancies in the US in 2012 (Martin et al. 2013) and only 1200 GCs that focus on prenatal care (NSGC 2014). With this in mind, there were seemingly contradictory responses regarding GCs roles in pre-test counseling (which, by definition is done in larger scale than post-test counseling): 38 % of GCs responded that pre-test counseling “should only be administered by GCs,” but nearly 67 % also agreed it could be administered by other health professionals and 31 % responded that an informational brochure or video was sufficient. The acceptability of non-formal genetic counseling may depend on the GCs perception of that professional’s or medium’s adequacy; further study would be illustrative.

Formal post-test genetic counseling for all individual positive results, as preferred by respondents, also requires substantial resources when considering that ECS panels can have individual positive rates of 23 % (Lazarin et al. 2013). ECS by next-generation sequencing confers even higher positive rates (Lazarin et al. 2014b). If all pregnant women underwent pre-test counseling and all positive results underwent post-test counseling, and a GC is necessary to perform this counseling, 1200 GCs would potentially be responsible for 5,000,000 consultations per year (4,000,000 pregnancies + 1,000,000 positive carrier results, if all pregnancies were screened).

Interestingly, a majority of obstetricians in the Benn et al. survey (2014) also responded that GCs should provide genetic counseling. Meeting the desire for formal genetic counseling, although it matches each individual patient with the optimal specialist, suffers from shortage of resources and necessitates alternative models (Minkoff and Berkowitz 2014) or substantial expansion of the number of RGCs.

The joint statement by Edwards et al. (2015) cites formal genetic counseling by a board-certified genetic counselor as indicated only in the case of two individuals identified as carriers for the same condition. This addresses the smallest but highest risk population. The joint statement also asserts that providers should establish a protocol for handling ECS results. Presumably, this can be done without formal genetic counseling for most cases, though an optimal and scalable protocol is yet to be established.

### Personal Preferences versus Professional Practices

Two survey sections addressed personal preferences and clinical practice. As previously stated, an overwhelming majority of GCs preferred to be screened for more diseases rather than less. In practice, 52 % of RGCs were not offering ECS to any patients and 40 % were offering it to less than one-third of patients. This discordance may be based on perspective (for example, having greater education in genetics may increase comfort level with obtaining more information), or they may be based on practical or logistical implementation barriers, such as availability of counseling time. We also noted that 43 % of RGCs (*n* = 61) agreed that, “all patients should be offered ECS.” Yet, only 6 RGCs responded that they were *actually* offering ECS to all patients.

Reasons for discrepancy between what one would make available to oneself versus one’s patients, and reasons for what GCs state *should* be done and what *is* done merit further study.

Gestational timing also affected offering rates. Unsurprisingly, there was a preference for screening at earlier stages – in rank order, those were preconception, first trimester and second trimester screening (90, 71 and 43 %, respectively).

We attempted to assess non-clinical factors that may affect practice. Availability of time is a concern – over 50 % of RGCs were concerned about the time necessary to explain screening results and/or follow up on testing protocols, such as sequence analysis.

Perceived patient’s SES may be another factor affecting screening practices for a minority of RGCs. While RGCs reported that they do not use perceived SES in determining clinical offerings in one question, less agreed with a separate statement that they offer all patients identical carrier screening options, regardless of SES. The effect of patients’ real or perceived SES status on counseling protocols, if any, has not been fully explored and may merit further study.

### Differences between GCs in Reproductive Settings and in Non-reproductive Settings

Differences in offering protocols are, of course, expected between counselors who see patients for reproductive planning and those who do not. However, we were interested to determine any statistically significant differences in opinions. In general, these were not observed between GCs practicing reproductive genetics and those in other specialties.

Exceptions to this were found in opinions regarding disease panel construction. GCs outside of the reproductive field were more receptive to diseases of any severity level and any prevalence. Although we did not assess the reasoning, it may be that consistent exposure to such diseases (e.g., in pediatric settings) may influence these opinions.

### Practical Implications and Study Limitations

We surveyed approximately 17 % of GCs working in reproductive settings. To our knowledge, this is the first and largest survey of GCs’ ECS perspectives, practices and knowledge.

Where statistical comparisons were made, they are limited by increased likelihood of chance findings due to multiple testing problems inherent with univariate statistical tests. Statistically significant results should therefore be interpreted with caution.

The survey covered many areas (a strength) but revealed the need for in-depth explorations before drawing definitive conclusions (a limitation). ECS has been rapidly developing - this survey was administered more than 2 years ago and since then, the authors observe that ECS has gained acceptance and utilization among GCs and non-GC healthcare providers. Therefore, a replication study may find differences in particular with regard to clinical practice and counseling considerations. For example, the ACMG statement on ECS was not published at the time of this survey.

GCs perspectives are useful for considering widespread opinions in the design of practice guidelines that can be based, in part, on professional opinions. For example, even though a minimum carrier frequency is often cited as disease inclusion criterion, less than half of GCs in the present study agreed that this should persist.

The responses also indicate areas of significant concern for GCs – in particular, the time and content of pre- and post-test counseling. These data, and those resulting from further study, may help to inform alternative models for delivering counseling, such as involvement by non-GCs, or use of print, video or Internet media.

### Conclusion and Research Recommendations

We found that in early 2012, approximately 2 years after initial clinical availability, GCs generally supported ECS, at least in theory. There were some varying opinions on diseases to be included, though current ACMG/ACOG guidelines were considered too restrictive. Informed consent components should focus on simplicity, including generic consent, and GCs performing most post-test consultations is desirable, though this raises questions of resource availability. We also found that although there were acceptance and strong personal interest in testing, actual clinical utilization was lagging. Since administration of the survey, ECS utilization has increased and statements from professional organizations have been issued. The authors plan to resurvey for comparison, which may reveal different findings, particularly in regard to utilization, and indicate changing dynamics in the GC profession.
